# Intraductal papillary mucinous neoplasm of the pancreas presenting as a giant abdominal mass: A case report

**DOI:** 10.1016/j.amsu.2022.103264

**Published:** 2022-01-19

**Authors:** Abdelhakim Harouachi, Marouane Harhar, Ayoub Kharkhach, Nabil Khomsi, Chafik Rhoul, Tariq Bouhout, Tijani EL. Harroudi, Badr Serji

**Affiliations:** a Surgical Oncology Department, Regional Oncology Center, Mohammed VI University Hospital, Oujda, Morocco; b Mohammed First University Oujda, Faculty of Medicine and Pharmacy Oujda, Oujda, Morocco

**Keywords:** Intraductal papillary mucinous neoplasm IPMN, Intestinal type, Abdominal mass, The portal vein, Splenopancreatectomy

## Abstract

**Introduction:**

Intraductal papillary mucinous neoplasms (IPMNs) constitute a group of rare conditions with a potential for malignant degeneration. The appearance of symptoms should suggest degeneration. This case demonstrates an unusual case of a patient presenting an intestinal type IPMN that was revealed by a large abdominal mass.

**Case report:**

47-year-old woman with a history of hydatid cyst of the liver. The patient was admitted to our hospital for management of large abdominal mass measuring 185 × 128*190 mm. Intra-operative findings showed a voluminous tumor, of approximately 20 cm in all its dimensions, with double solido-cystic component at the expense of the neck and the body of the pancreas. The patient underwent splenopancreatectomy. The histopathological examination confirmed the presence of intestinal type of IPMN of pancreas.

**Discussion:**

Acute pancreatitis is revealed in the majority of cases of IPMNs, related to duct obstruction by secreting mucus plug. IPMNs are rarely the cause of a large abdominal mass. They are cystic lesions of slow evolution, macroscopically visible and rarely macrocystic, unlike serous cystadenoma. The tumor size is a powerful indicator of the malignancy of IPMNs. The current definitive and ideal treatment for main duct and mixed type IMPNs is a surgical resection.

**Conclusion:**

IPMNs are a cystic lesion, rarely revealed by a large mass.

## Highlights

• IPMNs are rarely the cause of a large abdominal mass.
• The tumor size is a powerful indicator of the malignancy of IPMNs.
• The current definitive and ideal treatment for main duct and mixed type IMPNs is surgical resection.

## 1 Introduction and importance

Intraductal papillary mucinous neoplasms of the pancreas (IPMNs) constitute a group of rare conditions with a potential for malignant degeneration. These mucin-producing cystic entities present 1% of pancreatic exocrine tumors. According to their anatomic ductal involvement, IPMNs are classified into: main duct, branch duct and mixed type [1]. Their incidence is unknown.

Although histologically benign, IPNMs progress to pancreatic ductal adenocarcinoma. These cysts are often asymptomatic and more frequent at sixteen decade [2]. The appearance of symptoms should suggest degeneration. They are a rare cause of abdominal mass [3].

The diagnosis of IPMNs is typically established based on histopathological proof, rather than radiological imaging. Despite the lack of specific features, imaging remains essential in risk staging, description of anatomical site, and follow-up. A clear consensus on the management of IPMNs have developed, which can be simple monitoring up to radical treatment [4]. The aim of this study is to present an unusual case report of a giant intestinal type IPMN that was revealed by a large abdominal mass, related to the portal vein. To our best knowledge, few case of IPMN presented by a voluminous mass without an invasive malignant component was reported in the literature. This work has been reported in line with the SCARE 2020 Guideline [5].

## 2 Case presentation

A 47-years-old female patient, nulligravida, presented to our hospital chiefly complaining of painless abdominal mass that was gradually increasing in size over a period of 6 months. She reported anorexia and weight loss associated with the mass. The patient had a history of hydatid cyst of the liver and she underwent resection of the protruding dome at age 36 years. No other comorbidities were reported. She was a non-smoker with no drug use, and had no history of allergies. She had no family history of cancer, or psychological history.

During the physical examination, vital parameters were within normal limit. The abdominal examination has exhibited a well-defined, fixed with regard to the deep plan, non-pulsatile, left sided abdominal mass.

**Fig. 1:**
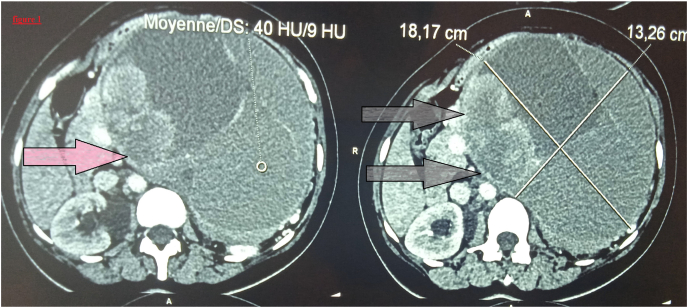
The computed tomography revealed a large left and paramedian intra peritoneal mass (arrow).

Abdominopelvic computed tomography CT scan showed a large left and paramedian intra peritoneal mass, of undetermined origin, roughly oval, well-defined, of mixed nature with a pure septale cystic component, mucinous impure cystic component and a solid component enhanced after injection of contrast, without presence of calcifications, measuring 185 × 128*190 mm. The mass pushes back left liver without any sign of noticeable invasion (Fig. 1). A recurrence of the hydatid cyst was suspected, also the gastric gastrointestinal stromal tumor. Her laboratory tests, including liver biochemical tests, routine blood examination, hydatid serology, and serum tumor markers, were all within normal limits.

**Fig. 2:**
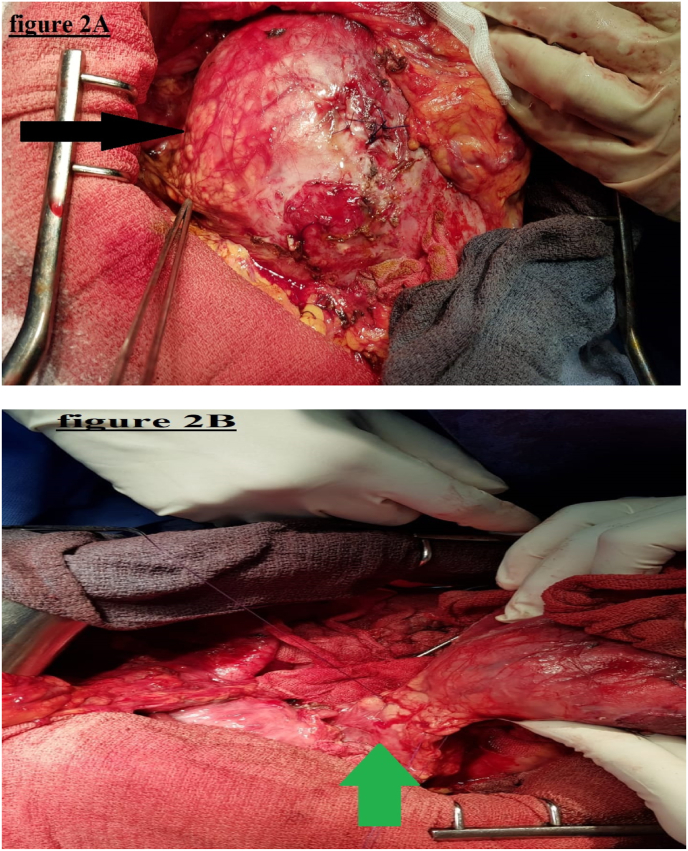
(2A and 2B): Intraoperative picture of a large mass at the expense of the neck and the body of the pancreas (black arrow) presenting contact with the portal vein (green arrow). (For interpretation of the references to colour in this figure legend, the reader is referred to the Web version of this article.)

Surgical approach was decided with an open laparotomy. The procedure was performed by a professor of surgery with over 15 years of surgical experience at an academic hospital. On exploration, a voluminous mass, of approximately 20 cm in all its dimensions, with double solido-cystic component at the expense of the neck and the body of the pancreas, closely related to the portal vein, without infiltration. There were no peritoneal implants, hepatic metastases, or adenomegaly. The tumor was firmly united by multiple adhesions with the adjacent structures, with displacement of the surrounding organs. Splenopancreatectomy involving the mass with omentectomy were performed after isolating the tumor from the portal vein and suture of a breach by single stitches prolene 5–0 (Fig. 2A, Fig. 2B). The operation time was 270 min, and the bleeding amount was 325 ml. Three units of packed red blood cells were transfused intra-operatively. A Penrose drain was placed in the cavity.

**Fig. 3:**
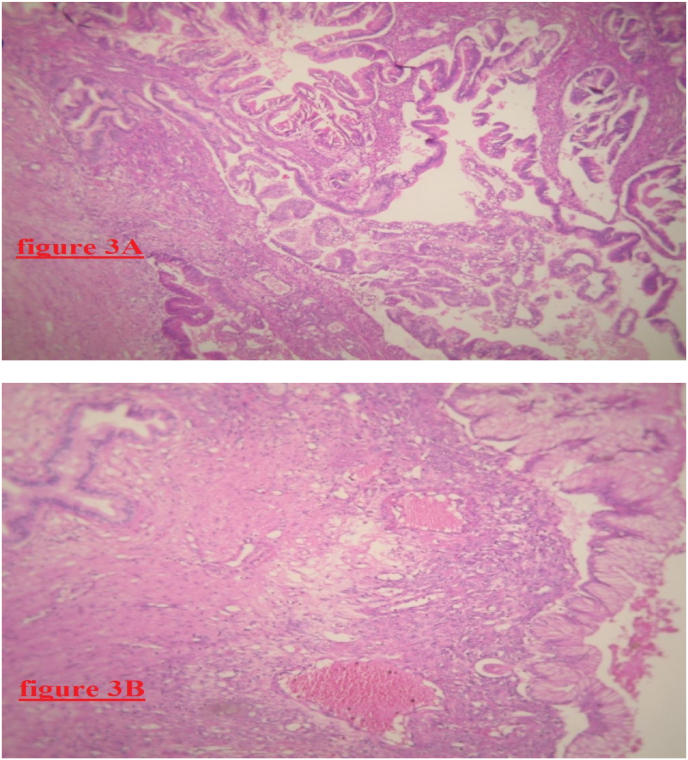
(3A and 3B): Microphotography of pancreatic patenchyma showing site of chronic pancreatitis and of intraductal papillary mucinous tumor proliferation without invasive malignant component.

Macroscopically, the specimen weighs 742g, ranges from 14 cm to 18.5 cm in size, poorly limited. It shows a solido-cystic, hemorrhagic, necrotic, friable, multilocular, and heterogeneous appearance. Histopathological examination of the specimen showed a pancreatic parenchyma, site of chronic pancreatitis and of intraductal mucinous tumor proliferation. The pancreatic ducts were largely ectatic lined by a unified or multi-layered muciparous cylindrical covering. The nuclei are sometimes regularly and sometimes atypical with a mitotic activity estimated at 5 mitoses per 10 pools of mucus resting a fibrous and very inflammatory chorion. This proliferation remains confined to the canal without any source of invasion (Fig. 3A, Fig. 3B). The immunohistochemistry staining was positive for anti-CK7, anti-CK20, and anti-CDX-2 antibodies. Twelve lymph nodes were analyzed that were all negative for malignancy. The diagnosis of intestinal type intraductal papillary mucinous neoplasm without invasive malignant component was established.

Postoperatively, the patient was doing well without any complications and she was discharged 14 days after the operation. After a follow-up of 6 months, the patient remains in good health, and there is no recurrence detected.

## 3 Clinical discussion

Intraductal papillary mucinous neoplasms IPMNs are the most common cystic tumors of the pancreas. They are characterized by the uncontrolled intraductal proliferation of the papillary-type epithelium due to accumulation of mucus leading to dilation and obstruction of the ducts, and the development of chronic pancreatitis lesions [4].

IPMNs are benign, precancerous lesions, located in the head of the pancreas in 60% of cases, and present a different degree of risk of malignant transformation to invasive ductal adenocarcinoma. They are observed at any age with a high incidence in the age group ranging from 60 to 65 years, constituting 15% of pancreatic tumors [6]. The association of intraductal papillary mucinous tumor of the pancreas with an invasive carcinoma has a major prognostic impact. Contrary to non-degenerate IPMNs which have a survival rate close to 100%, the 5-year survival rate is between 34% and 62% for IPMNs with an invasive component [4].

The location of the papillary proliferation and the morphology of the pancreatic ducts can be divided into 3 forms of IPMNs: “main duct type”, “brunch duct type”, and “mixed type”. The main duct and mixed type present a higher risk of dysplasia compared to branch duct type [7].

Four histological types depending on tissue architecture and cell differentiation: intestinal, pancreato-biliary, gastric, and oncocytic [8]. In our observation, the differentiation is of the intestinal type which defines a cell proliferation with the same histological profile of the intestinal cells. The intestinal type of IPMNs has a basophilic cytoplasm and cells that surround papillae forming large massifs protruding into the affected ducts. Mucus secretion is often at the apical pole, and the malignant transformation is a colloid type with indolent behavior. Regarding immunohistochemistry, the intestinal type of IPMNs is positive for MUC1, MUC5AC, and negative for MUC2 [8].

Risk stratification for IPMNs is assessed by main pancreatic duct dilation, cyst size, presence of wall nodules, or symptoms. Three categories of stratification can be distinguished: high risk of stigmata, lesions suspected of malignancy and “no risk” lesions. Surgical management is recommended in the event of a main pancreatic duct size greater than 10 mm, the presence of obstructive jaundice linked to a pancreatic head injury, the presence of a tissue component in the pancreatic ducts [4].

The clinical symptomatology of IMPNs is not specific. Its modes of revelation are variable. Acute pancreatitis is revealed in the majority of cases, related to duct obstruction by secreting mucus plug [9]. In terms of incidence, 12%_67% of IPMNs developed an acute pancreatitis [10].

The other main telltale symptoms are weight loss, diabetes, jaundice, and chronic pancreatitis. IPMNs are rarely the cause of a large abdominal mass as in our case [9]. They are cystic lesions of slow evolution, macroscopically visible (generally greater than 1cm), and rarely macrocystic, unlike serous cystadenoma [2,11]. A previous report stated that the tumor size is a powerful indicator of the malignancy of IPMNs. Navarro et al., determined in their retrospective study that the median benign tumor diameter is 2.80 cm versus 5.3 cm for malignant lesions [12]. We report in our case IPMN as large as 18 cm without an invasive malignant component.

In imaging, IPMNs are manifested on CT scan by dilation of the Wirsung's duct or secondary ducts without organic obstacles. They show cysts in a bunch of grapes. Intraductal and intracystic tumor adenoids can be observed, even calcifications on sections without injection of contrast product. CT is a modality of choice in assessing signs of degeneration [13,14]. In our case, abdomino-pelvic computed tomography scan has shown a large mass with a solido-cystic component pushing back the adjacent organs, adhering to the portal vein. The identification of the origin of the damage (isolated involvement of the main pancreatic duct or secondary ducts) in our observation is difficult by the usual radiological explorations.

The current definitive and ideal treatment for main duct and mixed type IMPNs is a surgical resection. Clinical and radiological monitoring is recommended in most cases of IMPNs-type brunch [4]. In our case, we realize splenopancreatectomy involving the mass. This choice is based on the size of the tumor.

## 4 Conclusion

IPMNs are a cystic lesion, having a significant malignant potential. Its classic appearance is generally small sized tumor, rarely revealed by a large mass without an invasive component.

## Declaration of competing interest

The authors declare that they have no conflict of interest.

## Sources of funding

The author(s) received no financial support for the research, authorship and/or publication of this article.

## Ethical approval

No ethical approval necessary.

## Consent

Written informed consent was obtained from the patient for publication of this case report and accompanying images. A copy of the written consent is available for review by the Editor-in-Chief of this journal on request.

## Author statement

**Dr Harouachi Abdelhakim:** Have written the article, have consulted the patient, prescribed all of the tests and prepared the patient for surgery and participated in the surgery.

**Dr Marouane Harhar:** have helped writing the article, data collection.

**Dr Kharkhach Ayoub:** have helped writing the article, data collection.

**Dr Nabil Khomsi:** have helped writing the article, data collection.

**Dr Chafik Rhoul:** have helped writing the article, data collection.

**Pr Bouhout Tariq** (oncology surgery professor): supervised the writing of manuscript.

**Pr El Harroudi Tijani** (oncology surgery professor): have supervised the writing of the paper.

**Pr Serji Badr** (oncology surgery professor): Writing, review and editing of the manuscript, and had been the leader surgeon of the case.

## Registration of research studies

Our paper is a case report; no registration was done for it.

## Guarantor

Harouachi Abdelhakim.

## Declaration of competing interest

The authors declared no potential conflicts of interests with respect to research, authorship and/or publication of the article.
